# Single-Strand Break End Resection in Genome Integrity: Mechanism and Regulation by APE2

**DOI:** 10.3390/ijms19082389

**Published:** 2018-08-14

**Authors:** Md. Akram Hossain, Yunfeng Lin, Shan Yan

**Affiliations:** Department of Biological Sciences, University of North Carolina at Charlotte, Charlotte, NC 28223, USA; mhossai5@uncc.edu (M.A.H.); ylin42@uncc.edu (Y.L)

**Keywords:** APE2, ATR-Chk1 DDR pathway, Genome integrity, SSB end resection, SSB repair, SSB signaling

## Abstract

DNA single-strand breaks (SSBs) occur more than 10,000 times per mammalian cell each day, representing the most common type of DNA damage. Unrepaired SSBs compromise DNA replication and transcription programs, leading to genome instability. Unrepaired SSBs are associated with diseases such as cancer and neurodegenerative disorders. Although canonical SSB repair pathway is activated to repair most SSBs, it remains unclear whether and how unrepaired SSBs are sensed and signaled. In this review, we propose a new concept of SSB end resection for genome integrity. We propose a four-step mechanism of SSB end resection: SSB end sensing and processing, as well as initiation, continuation, and termination of SSB end resection. We also compare different mechanisms of SSB end resection and DSB end resection in DNA repair and DNA damage response (DDR) pathways. We further discuss how SSB end resection contributes to SSB signaling and repair. We focus on the mechanism and regulation by APE2 in SSB end resection in genome integrity. Finally, we identify areas of future study that may help us gain further mechanistic insight into the process of SSB end resection. Overall, this review provides the first comprehensive perspective on SSB end resection in genome integrity.

## 1. Introduction

**Figure 1:**
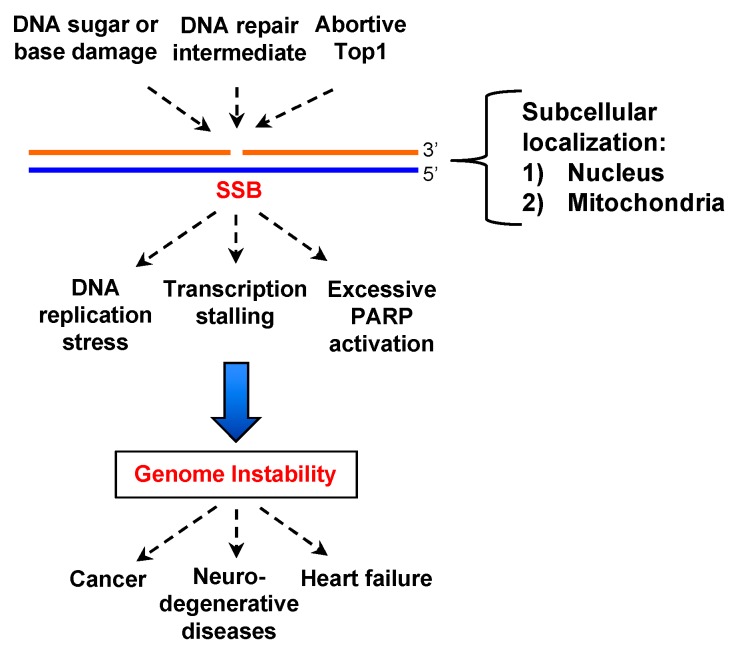
Generation and role of single-strand break (SSB) in genome integrity. SSBs may be derived from DNA sugar or base damage, defective DNA repair, and abortive Top1 activity, and are localized in nucleus and mitochondria. Unrepaired SSBs result in DNA replication stress, transcription stalling, and excessive PARP (Poly ADP-ribose polymerase) activation, leading to genome instability and human diseases such as cancer, heart failure, and neurodegenerative disorders.

DNA single-strand breaks (SSBs) are discontinuities in one strand of the DNA double helix, and are often associated with damaged or mismatched 5′- and/or 3′-termini at the sites of SSBs [1]. SSBs can arise from oxidized nucleotides/bases during oxidative stress, intermediate products of DNA repair pathways (e.g., base excision repair (BER)), and aborted activity of cellular enzymes (e.g., DNA topoisomerase 1) (Figure 1) [1,2]. Oxidative stress is an imbalance of the generation of reactive oxygen species (ROS) and anti-oxidant agents [2]. It has been estimated that more than 10,000 SSBs are generated per mammalian cell each day, representing the most common type of DNA lesions [3,4]. Unrepaired SSBs are localized primarily in nucleus and mitochondria and may result in DNA replication stress, transcriptional stalling, and excessive PARP activation, leading to genome instability (Figure 1) [1]. Accumulating evidence suggests that SSBs are implicated in the pathologies of cancer, neurodegenerative diseases, and heart failure (Figure 1) [1,2,5,6,7].

It is generally accepted that SSBs are repaired by various DNA repair mechanisms. Rapid global SSB repair mechanism includes SSB detection, DNA end processing, DNA gap filling, and DNA ligation, which is canonical SSB repair pathway [1]. The SSB repair pathway is sometimes considered as a specialized sub-pathway of BER [8]. Notably, PARP1 (Poly ADP ribose polymerase 1) and XRCC1 (X-ray repair cross-complementing protein 1) play essential roles in this canonical SSB repair pathway [9,10,11]. Alternatively, recent evidence shows that SSBs can also be resolved by either homologous recombination (HR) or alternative homologue-mediated SSB repair pathway [11,12]. Unrepaired SSBs during DNA replication can be converted to more deleterious DNA double-strand breaks (DSBs) [13]. The DNA replication-derived DSBs from SSBs result in chromosome breakages and translocations, leading to severe genome instability [14], although cohesion-dependent sister-chromatid exchange is available for repairing SSB-derived DSBs [15]. More details of various SSB repair pathways can be found from several recent reviews on the topic [8,11,16]. However, understanding how SSBs are generated, sensed, repaired, and signaled remains incomplete, largely because of the lack of efficient in vivo or in vitro experimental systems.

In this review, we will introduce a new concept “SSB end resection” in the field of genome integrity, and summarize the current molecular understanding of SSB end resection. We compare the major features of SSB end resection with DSB end resection. We then focus on the critical roles of SSB end resection in SSB signaling and repair. Finally, we identify several outstanding questions for future studies of SSB end resection. This perspective serves the first comprehensive review of SSB end resection for mechanistic studies on this topic in the field of genome integrity.

## 2. Concept of SSB End Resection

In general, SSB end resection is defined as the enzymatic end processing at SSB sites. The directionalities of SSB end resection include 3′ to 5′ direction and 5′ to 3′ direction, which are designated as 3′–5′ SSB end resection and 5′–3′ SSB end resection, respectively. After SSB end resection, an ssDNA (single-stranded DNA) gap of context-specific length is generated. Due to the technical difficulty in determining whether the resection of the two SSB ends are dependent on or mutually exclusive to each other, we cannot exclude the possibility of bidirectional SSB end resection; for simplicity, we propose the two possible SSB end resection with different directionality (i.e., 3′–5′ SSB end resection and 5′–3′ SSB end resection).

Recent studies are in support of the concept of 3′–5′ SSB end resection. It has been demonstrated that oxidative DNA damage-derived indirect SSBs are processed by APE2 (AP endonuclease 2, also known as APEX2 or APN2) in the 3′ to 5′ direction to promote ATR-Chk1 DNA damage response (DDR) pathway in *Xenopus* cell-free egg extract system [17,18]. Interestingly, it has also been shown that a defined site-specific SSB structure can be resected in the 3′ to 5′ direction by APE2 in *Xenopus* system and reconstitution experimental system [19]. Importantly, it was recently demonstrated that a 9nt-gap is formed in the 5′ side of a defined SSB structure for subsequent DNA repair in living cells [20]. These findings are consistent with the critical roles of 3′–5′ SSB end resection in genome integrity.

Some DNA metabolism enzymes, such as TDP2 (Tyrosyl-DNA phosphodiesterase 2) and APTX (Aprataxin), may digest SSB end in the 5′ to 3′ direction, suggesting a possible mechanism of 5′–3′ SSB end resection [8,21]. However, there are almost no in-depth studies showing whether and how the 5′–3′ SSB end resection happens. Thus, the potential biological or physiological relevance of 5′–3′ SSB end resection remains unclear. The long-patch BER pathway involves PCNA (Proliferating cellular nuclear antigen)-mediated DNA repair synthesis and FEN1 (Flap structure-specific endonuclease 1)-mediated degradation of a DNA strand [22], which is excluded from our defined 5′–3′ SSB end resection. Future investigations are still needed to test whether SSB end can be resected in the 5′ to 3′ direction in various different model systems. Thus, we focus on the 3’–5’ SSB end resection processes in this review.

**Figure 2:**
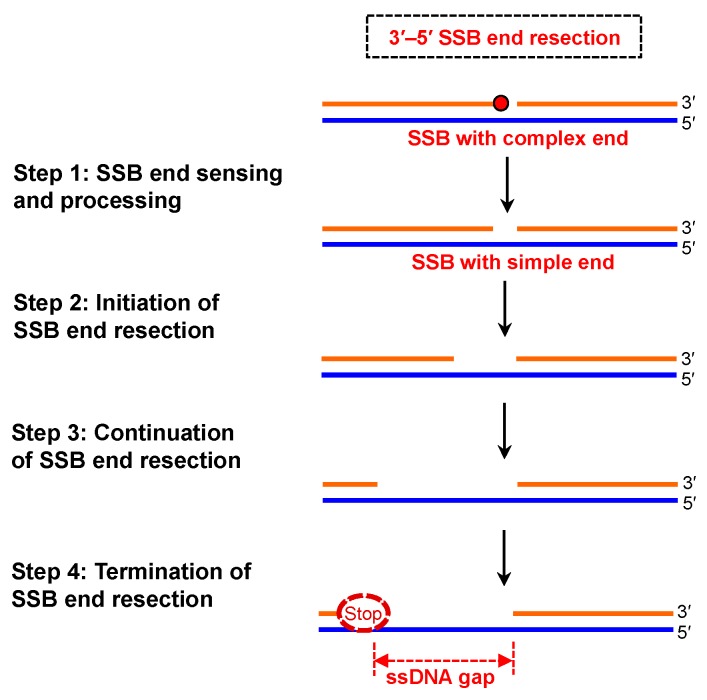
Proposed four steps of 3′–5′ SSB end resection: End sensing and processing, initiation, continuation, and termination of SSB end resection.

Here, we propose a four-step molecular mechanism involved in the processes of 3′–5′ SSB end resection (Figure 2): (I) Step 1 is SSB end sensing and processing; (II) Step 2 is the initiation phase of SSB end resection; (III) Step 3 is the continuation phase of SSB end resection; and (IV) Step 4 is the termination of SSB end resection. In the next section, we delineate the details of these four steps for SSB end resection.

## 3. Molecular Mechanism of SSB End Resection

### 3.1. SSB End Sensing and Processing

During the canonical SSB repair, SSB end sensing by sensor protein such as PARP1 is critical for the subsequent DNA repair process [1,23]. SSBs with the “-OH” groups at both ends are designated as SSBs with simple ends. On the other hand, SSBs with chemically heterogeneous structures, such as 3′-Top1 adduct, 3′-phosphate, 3′-phosphoglycolate, 5′-Top2 adduct, 5′-aldehyde, 5′-deoxyribose phosphate, or 5’-adenylate (AMP), are designated as SSBs with complex ends [8,21]. These complex ends of SSBs are recognized and processed or removed by various DNA metabolism enzymes such as TDP1 (Tyrosyl-DNA phosphodiesterase 1), APE1 (AP endonuclease 1), Polymerase beta, FEN1, and APTX, among others [8,21]. Such SSB end processing is important for canonical SSB repair pathway. However, it remains unclear how cells decide to proceed with the canonical SSB repair pathway, or alternatively, the SSB end resection-mediated non-canonical SSB repair pathway. Mechanistic studies are needed to find out whether the SSB end processing is critical for making decisions on choice of various SSB repair pathways.

### 3.2. Initiation of SSB End Resection

It is critical for cells to resect SSBs in the 3′–5′ direction only when necessary, leading to a ssDNA gap. However, such a ssDNA gap is more deleterious than just a nick or 1-nt gap in genome. Thus, this initiation phase of SSB end resection must be highly regulated via essential regulatory mechanisms. It has been demonstrated that several DNA metabolism enzymes may resect SSBs to initiate the SSB end resection process in vitro.

DNA exonucleases such as APE2, APE1, and Mre11 may be involved in SSB end resection initiation. APE2 has strong 3′–5′ exonuclease activity but weak AP endonuclease activity [24,25]. It has been shown that APE2 resects ~3nt on a defined SSB structure in the 3′–5′ direction even in the absence of PCNA in vitro [24]. Notably, ~1–4 nt ssDNA gap structure will significantly enhance APE2’s 3′–5′ exonuclease activity in vitro [24]. However, a defined SSB structure is still resected into ~1–3 nt ssDNA gap in the 3’–5’ direction when APE2 is absent in *Xenopus* cell-free system [19]. These observations suggest that APE2 may contribute to the initiation of SSB end resection in in vitro assays, or alternatively, that other exonuclease resects SSB in the absence of APE2 using cell-free egg extracts. Considering the requirement of ssDNA for APE2’s PCNA-mediated 3′–5′ exonuclease activity, APE2 may not be the exonuclease to initiate the SSB end resection process in vivo. Therefore, it remains unclear whether APE2 initiates SSB end resection in in vivo systems. APE1 (AP endonuclease 1, also known as Ref-1 or APN1) has weak 3′–5′ exonuclease activity but strong AP endonuclease activity [26]. It has been demonstrated that APE1 can resect a SSB structure into ~1–3nt ssDNA gap structure in the 3′ to 5′ direction in vitro [19,27]. Of note, APE1 can also resect 1-nt gap or 2-nt gap structures in vitro. APE1’s 3′–5′ exonuclease activity was shown to prevent trinucleotide repeat expansions [28]. APE1 is also shown to remove mismatches at the 3′-end of SSB site [29]. Interestingly, APE1 mutants at the F266 and W280 residues significantly enhance its 3′–5′ exonuclease activity [30]. Structure determinant of such APE1’s 3′–5′ exonuclease activity from a SSB structure has been recently elucidated in more details [31]. However, it remains elusive whether APE1 resects SSB in the 3′ to 5′ direction in vivo. Furthermore, Mre11’s exonuclease from the Mre11-Rad50-Nbs1 (MRN) complex can resect SSB with simple end in the 3′–5′ direction in reconstitution system with purified proteins [32]; however, the potential role of Mre11 in SSB end resection initiation requires a nearby DSB end [33,34]. Future studies are needed to determine whether SSB structure without a nearby DSB can be resected by Mre11’s 3′–5′ exonuclease activity.

Furthermore, other type of DNA metabolism enzymes such as helicase and endonuclease may also be involved in the SSB end resection initiation. It has been reported that a 9-nt ssDNA gap is formed in the 3′–5′ direction of an oxidative or alkylation lesion in living cells [20]. Mechanistic studies have revealed that the ssDNA gap formation is mediated by DNA helicase RECQ1 and endonuclease ERCC1-XPF in cooperation of PARP1 and RPA, and that the ssDNA gap formation in the 5′ side of DNA lesion promotes subsequent DNA repair [20]. Consistent with this observation, Rad1-Rad10 nuclease in budding yeast (counterpart of human ERCC1-XPF) can remove 3’ complex end of SSB and further resect SSB several nt in the 3′–5′ direction to promote the repair of hydrogen peroxide-induced SSBs [35]. In addition, it is also possible that some previously unidentified DNA exonucleases and helicases/endonucleases can initiate the SSB end resection. Unbiased *de novo* identification and functional characterization of these DNA metabolism enzymes are needed to reveal more molecular details in the initiation phase of SSB end resection.

### 3.3. Continuation of SSB End Resection

After the initiation phase, SSB end resection processing is continued by DNA metabolism enzymes with higher processivity of 3′–5′ exonuclease activities. The first important player in continuation of SSB end resection is APE2, which has strong PCNA-mediated 3′–5′ exonuclease activity. Since the apparent outcome of SSB end resection is to generate a longer stretch of ssDNA gap, the APE2-mediated SSB end resection continuation must be under tight regulations, and such 3′–5′ SSB end resection only happens when it is necessary. At least three different types of regulatory mechanisms have been suggested to determine how APE2 contributes to SSB end resection continuation:

The first regulatory mechanism is to regulate how APE2 is recruited to SSB sites. APE2 is localized in nucleus and mitochondria [36], although there is no report on underlying mechanism of how exactly APE2 is imported into these organelles, respectively. APE2 interacts with PCNA via APE2’s PIP (PCNA-interacting protein) box and PCNA’s IDCL (interdomain connector loop) motif, which is designed as the first mode of APE2-PCNA interaction and is critical for the recruitment of APE2 to oxidative stress-damaged chromatin DNA [17,24,25,37,38]. Therefore, PCNA may play an important role in the recruitment of APE2 to SSB sites on damaged chromatin.

The second regulatory mechanism is to enhance APE2’s 3′–5′ exonuclease activity via APE2 interaction with ssDNA. APE2 interaction with PCNA is not sufficient for promoting its 3′–5′ exonuclease activity. Recent studies have demonstrated that a unique Zf-GRF motif within APE2 C-terminus plays an essential role for its recognition and binding to ssDNA region and associated 3′–5′ exonuclease activity [18]. Once APE2 Zf-GRF interacts with ssDNA, the conformation of the catalytic domain within APE2 N-terminus may be changed for maximum 3′–5′ exonuclease activity. More structure/function analysis is needed to clarify how such ssDNA interaction within APE2 Zf-GRF promotes its 3′–5′ exonuclease activity.

The third regulatory mechanism is to promote APE2’s 3′–5′ exonuclease activity via two distinct modes of the APE2-PCNA interaction. In addition to the first mode of APE2-PCNA interaction, APE2 Zf-GRF motif interacts with PCNA’s C-terminus, which is designated as the second mode of APE2-PCNA interaction [19]. Several separation-of-function mutants within APE2 have been characterized in *Xenopus* APE2 to distinguish the two modes of APE2-PCNA interaction [19]. Notably, the two modes of APE2-PCNA interaction are neither dependent on nor exclusive to each other. Both modes of APE2-PCNA interaction are critical to promote APE2’s 3′–5′ exonuclease activity in *Xenopus* [19]. Similarly, yeast APE2 binds to the PCNA IDCL motif and C-terminus to enhance its 3′–5′ exonuclease activity [25]. Based on the high similarity within APE2 Zf-GRF region among different species, the critical role of the second mode of APE2-PCNA interaction for APE2’s exonuclease activity is likely conserved in mammalian cells.

Notably, APE2’s 3′–5′ exonuclease activity has been demonstrated and characterized in vitro from experimental model organisms including *Arabidopsis thaliana*, *Trypanosoma cruzi*, *Ciona intestinalis*, *Saccharomyces cerevisiae*, *Schizosaccharomyces pombe*, *Xenopus laevis*, and *Homo sapiens* [19,36,39,40,41,42,43], suggesting that the role of APE2’s 3′–5′ exonuclease activity in SSB end resection is highly conserved during evolution. APE2 3′–5′ exonuclease activity is important for the removal of 3′-blocked termini to repair DNA lesions from hydrogen peroxide treatment in *Saccharomyces cerevisiae* [38]. In addition, *Ciona intestinalis* APE2 has 3′–5′ exonuclease activity and contributes to protection and survival from oxidative stress [43]. Human APE2 is mostly localized in the nuclei and to some extent in the mitochondria [36]. Furthermore, PCNA interacts with human APE2 to stimulate APE2’s 3′–5′ exonuclease activity, which is important for removing 3′-end adenine opposite from 8-oxoG (7,8-dihydro-8-oxoguanine) and subsequent 3′–5′ end resection [37]. Notably, oxidative stress also promotes the colocalization of APE2 with PCNA in living cells [37].

### 3.4. Termination of SSB End Resection

The apparent outcome of the 3′–5′ SSB end resection is DNA strand degradation, making it deleterious for genome stability if the 3′–5′ SSB end resection is not terminated when necessary. In vitro data have shown that a 3′ recessed ssDNA/dsDNA structure can be resected almost completely by PCNA-mediated APE2’s 3′–5′ exonuclease activity [18]. It is reasoned that some regulatory mechanisms are necessary to negatively regulate APE2’s 3′–5′ exonuclease activity in vivo once sufficient ssDNA gap is generated. More studies are needed to dissect the molecular details of how SSB end resection is terminated.

## 4. SSB End Resection and DSB End Resection

**Figure 3:**
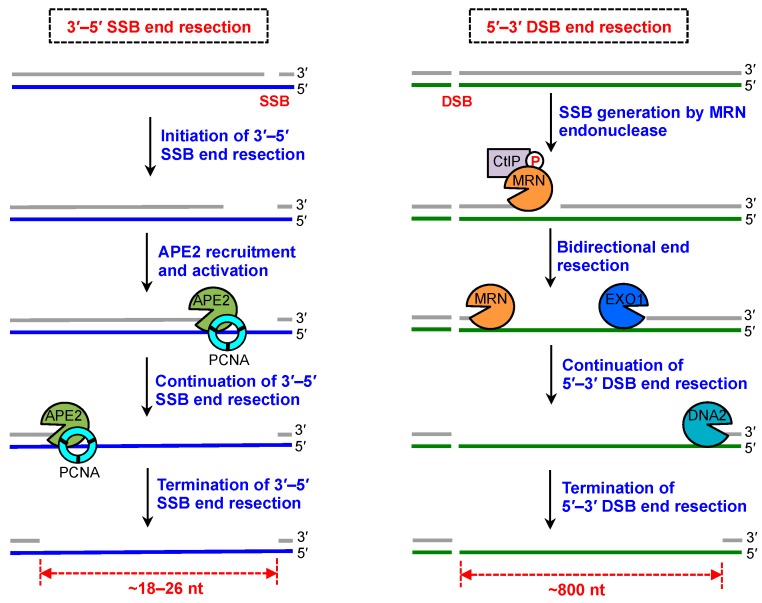
SSB end resection and DSB end resection. Left panel shows molecular details of 3′–5′ SSB end resection. Following initiation of 3′–5′ SSB end resection via an unknown mechanism, APE2 is recruited and activated by PCNA interaction and ssDNA association. The 3′–5′ SSB end resection is continued by APE2’s 3′–5′ exonuclease activity to generate ssDNA (~18–26 nt) and terminated by an unknown mechanism. The right panel shows molecular details of 5′–3′ DSB end resection. DSB end is recognized by MRN complex and nicked by CtIP-mediated Mre11’s endonuclease activity, followed by bidirectional end resection through 3′–5′ exonuclease activity of the MRN complex and 5′–3′ exonuclease activity of EXO1 (Exonuclease 1). The 5′–3′ DSB end resection is continued by DNA2 to generate a longer stretch of ssDNA (~800 nt).

It is well documented that DSB end resection in the 5′–3′ direction is critical for DSB repair and DSB signaling [44,45,46]. Notably, Mre11 contributes to DNA repair of DSB ends or protein-DNA crosslinks via its 3′–5′ exonuclease activity [32,47]. Accumulating evidence suggests that Mre11’s endonuclease activity is required for generating SSBs at DSB ends, which are further resected by 3′–5′ exonuclease activity of the MRN complex and 5′–3′ exonuclease activity of EXO1 (Exonuclease 1) (Figure 3) [33,34,48]. This mechanism is designated as bidirectional DSB end resection [49]. After EXO1’s initial end resection, the 5′–3′ DSB end resection is further continued by other DNA metabolism enzymes such as DNA2, which is known as the two-step mechanism for 5′–3′ DSB end resection (Figure 3) [50].

**Figure 4:**
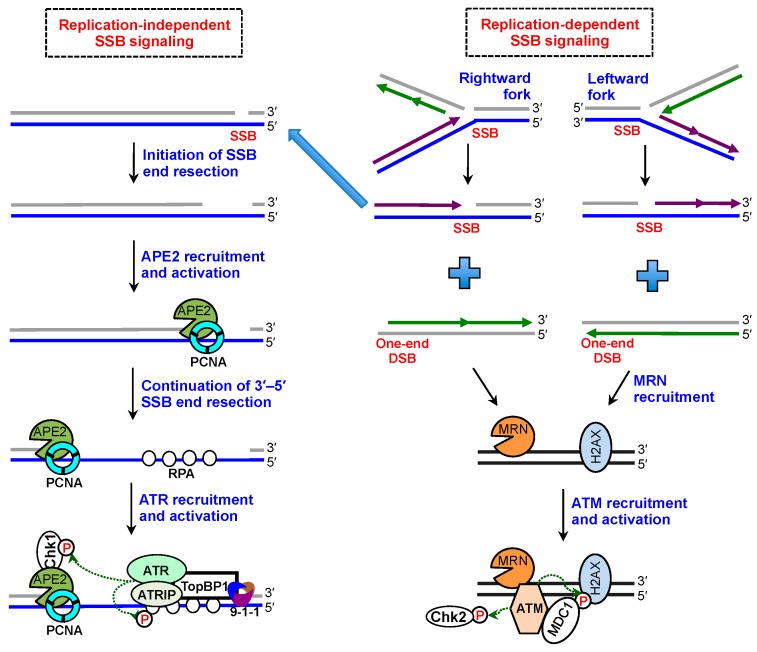
SSB signaling. Left panel demonstrates replication-independent SSB signaling. Following initiation of SSB end resection, APE2 is recruited and activated by PCNA interaction and ssDNA association. SSB end is resected by APE2 in the 3′–5′ direction to generate ssDNA for RPA recruitment and assembly of ATR DDR protein complex including ATR, ATRIP, TopBP1, and the 9-1-1 complex. Activated ATR phosphorylates Chk1 and RPA32. Right panel shows replication-dependent SSB signaling. When replication fork (rightward or leftward) meets SSB site, one-end DSB and new SSB are generated. The replication-derived SSB may proceed with 3′–5′ SSB end resection and subsequent ATR DDR pathway. The one-end DSB triggers MRN complex recruitment and ATM DDR activation including γ–H2AX and Chk2 phosphorylation.

In contrast to DSB end resection, SSB end resection has several distinctive features. First, the directionality of SSB end resection is in the 3′ to 5′ direction, whereas overall DSB end resection is in the 5′ to 3′ direction. It remains unclear whether a SSB can be resected in the 5′ to 3′ direction. The functionalities of DNA end resection are likely through different DNA metabolism enzymes involved in the two different DNA end resection (i.e., DSB and SSB end resection) pathways. Second, the ssDNA gap generated after 3′–5′ SSB end resection is relatively short (~18–26 nt), whereas the ssDNA after DSB end resection is large (~800 nt) (Figure 3) [19,48]. Reconstitution evidence suggests that CtIP/Sae2 promotes endonuclease activity within Mre11 to make SSB from a nearby protein-occluded DSB end [51,52]. Although the size of ssDNA region generated from DSB end resection or SSB end resection is different, the RPA-coated ssDNA serves the platform for assembly of the DDR protein complex to trigger ATR-Chk1 DDR pathway activation (Figure 4). Third, the 3′–5′ SSB end resection near a DSB end requires Mre11 exonuclease activity, whereas 3′–5′ SSB end resection without a nearby DSB end requires APE2 exonuclease activity (Figure 3) [19,53]. Interestingly, it appears that while APE2 and Mre11 have related functions, they cannot compensate for the absence of each other in regards to 3′–5′ SSB end resection with or without DSB end, respectively.

## 5. Roles of SSB End Resection in SSB Signaling, SSB Repair, and Beyond

The 3′–5′ SSB end resection mediated by APE2 is essential for the ATR-Chk1 DDR pathway following oxidative stress in *Xenopus* egg extracts [17,18,54,55,56,57]. Especially, APE2’s 3′–5′ exonuclease activity is particularly critical for oxidative stress-induced DDR pathway activation [17]. Furthermore, a defined site-specific SSB structure triggers ATR-Chk1 DDR pathway in a DNA replication-independent fashion in *Xenopus* egg extracts [19]. Notably, APE2’s 3′–5′ exonuclease activity is essential for the defined SSB-induced ATR-Chk1 DDR pathway, whereas CDK (Cyclin-dependent kinase) kinase activity is dispensable for the SSB-induced DDR pathway [19]. Furthermore, the APE2-mediated 3′–5′ SSB end resection is required for ssDNA generation and assembly of ATR, ATRIP, TopBP1, and the 9-1-1 (Rad9-Rad1-Hus1) complex onto SSB sites to trigger the ATR-Chk1 DDR pathway (Figure 4) [19]. Therefore, the APE2-mediated 3′–5′ SSB end resection is essential for SSB signaling.

Oxidative DNA damage-derived SSBs can trigger ATM-Chk2 DDR pathway activation in mammalian cells [58]. Although unrepaired SSBs in XRCC1-deficient cells trigger ATM activation to prevent the generation of DSBs, the underlying mechanism of SSB-induced ATM DDR pathway activation remains unclear. It has been shown that one-end DSB is generated when DNA replication fork meets with SSB (Figure 4) [13]. Furthermore, replication-derived BER-processed SSBs from methylation damage can trigger checkpoint signaling such as γ-H2AX, leading to chromatid breaks and chromosome translocations [14]. It is conceivable that replication-derived DSBs from SSBs can activate the ATM-Chk2 DDR pathway (Figure 4). In addition, such DSBs from SSBs during replication can be repaired by cohesion-dependent sister-chromatid exchange [15].

What is the role of 3′–5′ SSB end resection for SSB repair? XRCC1-mediated canonical SSB repair have been revealed in reconstitution system with recombinant human proteins or cultured mammalian cells [59,60]. XRCC1 promotes PNKP (Polynucleotide kinase phosphatase)-mediated SSB end termini processing, followed by gap filling by DNA polymerase beta and sealing by DNA ligase 3. More details of the canonical SSB repair can be found from recent comprehensive reviews [1,11]. It has been demonstrated that defined plasmid-based SSB structure is repaired in about 30 min in the *Xenopus system* [19]. Because of the involvement of 3′–5′ SSB end resection, this distinct repair pathway is designated as non-canonical SSB repair. The capacity of SSB repair in *Xenopus* system is comparable to that characterized in human SSB repair systems [59]. However, it remains unclear how the XRCC1-mediated canonical SSB repair pathway and the APE2-mediated non-canonical SSB repair pathway contribute to the overall SSB repair. Do they work coordinately or independently? Notably, one distinct feature of the non-canonical SSB repair pathway is dependence on the ATR-Chk1 DDR pathway activation in *Xenopus* egg extracts [19]. Although the precise mechanism underlying the non-canonical SSB repair remains to be elucidated, we speculate two possible mechanisms: one or more SSB repair regulators are phosphorylated by ATR or Chk1 kinases, which are required for promoting SSB repair; alternatively, efficient SSB repair is suppressed by an inhibitory factor that can be phosphorylated by ATR or Chk1 to relieve the suppression. Although it is currently unknown whether the role of APE2 in SSB end resection-mediated non-canonical SSB repair is conserved in mammalians, previous studies have shown that APE2 is important for overall SSB repair following oxidative stress in mammalian cells [36,37]. The APE2-mediated non-canonical SSB repair in *Xenopus* has important implications in mammalian cells, especially in terminally differentiated cells such as neuron cells, most of which remain in the G0 or G1 phase of the cell cycle.

Unrepaired SSBs are implicated in human diseases such as neurodegenerative disorders, cancer, and heart failure [1,5,6,7]. SSB repair has been associated with hereditary genetic diseases including Ataxia-oculomotor apraxia 1 (AOA1) and spinocerebellar ataxia with axonal neuropathy 1 (SCAN1) [1]. Both germline and tumor-associated variants of genes encoding SSB repair proteins (e.g., XRCC1, APE1, and Polymerase beta) have been identified in humans, suggesting SSB repair as a tumor suppressor mechanism [61]. However, it remains unclear whether the SSB-induced SSB signaling or the associated SSB repair pathways play direct or indirect roles in tumorigenesis. It has been shown recently in senescent epithelial cells that ROS induces more SSBs and downregulates PARP1 expression, leading to defective SSB repair and emergence of post-senescent transformed and mutated precancerous cells [5]. Thus, the mutagenicity of accumulated unrepaired SSBs in epithelial cells is proposed as the driver of cancer development [62]. APE2 mutants have been found in several cancer patients, suggesting that defective SSB end resection is implicated in cancer development [19]. Future mechanistic studies using mammalian cell lines and genetically engineered mouse models will allow us to better understand how APE2-medaited SSB end resection is involved in cancer development. Interestingly, accumulation of SSBs was found in cardiomyocytes of the failing heart and unrepaired SSB triggers DDR pathway, and increases inflammatory response through NF-κB signaling [6]. In addition, newly defined distinct homology-dependent SSB repair pathways are proposed to support gene correction or editing using ssDNA donors at sites of SSBs [12,63]. The initiation of HR at SSBs is distinct from HR from DSB sites, and a current understanding of SSB-induced HR is summarized in a recent review [16]. Together, findings from SSB end resection studies will contribute to the field of genome integrity. 

## 6. Concluding Remarks and Perspectives for Future Studies

Although we have just begun to understand role and mechanism of SSB end resection in genome integrity, many significant questions in studies of SSB end resection remain unanswered. What is the molecular mechanism underlying the initiation phase of SSB end resection in vivo? Although a few DNA nucleases demonstrate 3′–5′ exonuclease activity in vitro, it is vital to determine how SSB end resection is initiated exactly. We speculate that type and complexity of SSB ends (e.g., simple ends or complex ends) may be important for the initiation of SSB end resection. How is SSB end resection terminated or negatively regulated? Whereas SSB end resection initiates and continues in the 3′–5′ direction, leading to ssDNA generation, some regulatory mechanisms should be in place to terminate SSB end resection when necessary. Otherwise, a longer stretch of ssDNA will be generated, leading to more severe genome instability such as DSBs and chromosome translocations. How does cell make decisions to repair SSBs via canonical or non-canonical SSB repair pathway? We speculate that most SSBs are repaired via XRCC1-mediated canonical SSB repair pathway, and that APE2-mediate non-canonical SSB repair may take place only when the amount of SSBs is more than a repair threshold. Although non-canonical SSB repair requires ATR DDR pathway [19], future work is still needed to reveal molecular details of this non-canonical SSB repair. Will nucleosome and chromatin remodeling complex at or near SSB sites regulate SSB end resection? A recent report has demonstrated that PARP3 recognizes site-specific SSB in nucleosome and monoribosylates Histone 2B in DT40 cells [64]. It is also shown that SNF2 chromatin remodeling protein ALC1 is important for chromatin relaxation and SSB repair [65]. Thus, it is important to determine whether SSB end resection is regulated by the context in chromatin including nucleosome and chromatin remodeling complex.

Various experimental systems including the *Xenopus* egg extract system and mammalian cells in culture have been developed to study SSB repair and signaling. The *Xenopus* egg extracts system has been utilized and optimized to dissect different aspects of SSB end resection directly: hydrogen peroxide-induced indirect SSBs on chromatin DNA in *Xenopus* low speed supernatant and defined site-specific plasmid-based SSBs in *Xenopus* high-speed supernatant [17,18,19]. Thus, *Xenopus* egg extracts system provides an excellent experimental system to reveal the molecular details of replication-dependent and -independent SSB end resection in SSB repair and signaling. In addition, SSBs are accumulated after treatment of DNA damaging agents (e.g., methyl methanesulfonate and hydrogen peroxide) in mammalian cells such as terminally differentiated muscle cells and cardiomyocyte [6,66]. SSBs are also generated when BER proteins such as XRCC1 is knocked down or deficient in mammalian cells [6,58]. SSBs, but not DSBs, can be induced after local UVC irradiation in XPA-UVDE cells which express UV damage endonuclease (UVDE), but which are deficient in nucleotide excision repair protein XPA [67,68]. Site-specific SSB can be introduced by transient transfection with Cas9 and gRNA expression vectors in human osteosarcoma cells (U2OS-DR-GFP) and mouse embryonic stem cells (ES-DR-GFP) harboring a single genetically integrated copy of the DR-GFP reporter [16,69]. A recent study has demonstrated that a small ssDNA gap is generated in the 5’ side of SSB in plasmid-transfected cells, suggesting that the 3′–5′ SSB end resection is conserved in mammalian cells [20]. Future studies of the outstanding questions using these various experimental systems will provide a better understanding of all aspects of SSB end resection in genome integrity.

Taking together, we introduce the concept and mechanism of SSB end resection and summarize the current understanding on the biological significance of SSB end resection in genome integrity.

## Abbreviations

9-1-1 complex	Rad9-Rad1-Hus1 complex
8-oxoG	7,8-dihydro-8-oxoguanine
APE1	AP endonuclease 1
APE2	AP endonuclease 2
APTX	Aprataxin
BER	Base excision repair
CDK	Cyclin-dependent kinase
DDR	DNA damage response
DSB	Double-strand break
EXO1	Exonuclease 1
FEN1	Flap structure-specific endonuclease 1
HR	Homologous recombination
IDCL motif	Interdomain connector loop motif
ROS	Reactive oxygen species
SSB	Single-strand break
PARP1	Poly ADP ribose polymerase 1
PARP3	Poly ADP ribose polymerase 3
PCNA	Proliferating cellular nuclear antigen
PIP box	PCNA-interacting protein box
PNKP	Polynucleotide kinase phosphatase
ssDNA	Single-stranded DNA
TDP1	Tyrosyl-DNA phosphodiesterase 1
TDP2	Tyrosyl-DNA phosphodiesterase 2
UVDE	UV damage endonuclease
XRCC1	X-ray repair cross-complementing protein 1
